# Supplementary material for: Bridging between material properties of proteins and the underlying molecular interactions
Source: PLoS One. 2021 May 5;16(5):e0247147. doi: 10.1371/journal.pone.0247147 (PMC8099097; doi:10.1371/journal.pone.0247147)
Supplement: Attachment — Submitted filename: responsesToReviewers.pdf [file pone.0247147.s001.pdf]

Dear Editor,

We are grateful for the reviewers' valuable feedback and comments. We have revised the manuscript accordingly. Below are our point-by-point responses.

Thank you,

The Author

PONE-D-21-02708
Bridging Between Material Properties Of Proteins And The Underlying Molecular Interactions
PLOS ONE

Dear Dr. Song,

Thank you for submitting your manuscript to PLOS ONE. After careful consideration, we feel that it has merit but does not fully meet PLOS ONE's publication criteria as it currently stands. Therefore, we invite you to submit a revised version of the manuscript that addresses the all the points raised by both reviewers.

Please submit your revised manuscript by Apr 26 2021 11:59PM. If you will need more time than this to complete your revisions, please reply to this message or contact the journal office at plosone@plos.org. When you're ready to submit your revision, log on to https://www.editorialmanager.com/pone/ and select the 'Submissions Needing Revision' folder to locate your manuscript file.

Please include the following items when submitting your revised manuscript:

- A rebuttal letter that responds to each point raised by the academic editor and reviewer(s). You should upload this letter as a separate file labeled 'Response to Reviewers'.
- A marked-up copy of your manuscript that highlights changes made to the original version. You should upload this as a separate file labeled 'Revised Manuscript with Track Changes'.
- An unmarked version of your revised paper without tracked changes. You should upload this as a separate file labeled 'Manuscript'.

If you would like to make changes to your financial disclosure, please include your updated

statement in your cover letter. Guidelines for resubmitting your figure files are available below the reviewer comments at the end of this letter.

If applicable, we recommend that you deposit your laboratory protocols in protocols.io to enhance the reproducibility of your results. Protocols.io assigns your protocol its own identifier (DOI) so that it can be cited independently in the future. For instructions see: http://journals.plos.org/plosone/s/submission-guidelines#loc-laboratory-protocols

We look forward to receiving your revised manuscript.

Kind regards,

Claudio M. Soares, Ph.D
Academic Editor
PLOS ONE

Journal Requirements:

When submitting your revision, we need you to address these additional requirements.

1. Please ensure that your manuscript meets PLOS ONE's style requirements, including those for file naming. The PLOS ONE style templates can be found at

https://journals.plos.org/plosone/s/file?id=wjVg/PLOSOne_formatting_sample_main_body.pdf and

https://journals.plos.org/plosone/s/file?id=ba62/PLOSOne_formatting_sample_title_authors_affiliations.pdf

[Note: HTML markup is below. Please do not edit.]

Reviewers' comments:

Reviewer's Responses to Questions

Comments to the Author

1. Is the manuscript technically sound, and do the data support the conclusions?

The manuscript must describe a technically sound piece of scientific research with data that supports the conclusions. Experiments must have been conducted rigorously, with appropriate controls, replication, and sample sizes. The conclusions must be drawn appropriately based on the data presented.

Reviewer #1: Partly

Reviewer #2: No

---

2. Has the statistical analysis been performed appropriately and rigorously?

Reviewer #1: Yes

Reviewer #2: Yes

---

3. Have the authors made all data underlying the findings in their manuscript fully available?

The PLOS Data policy requires authors to make all data underlying the findings described in their manuscript fully available without restriction, with rare exception (please refer to the Data Availability Statement in the manuscript PDF file). The data should be provided as part of the manuscript or its supporting information, or deposited to a public repository. For example, in addition to summary statistics, the data points behind means, medians and variance measures should be available. If there are restrictions on publicly sharing data—e.g. participant privacy or use of data from a third party—those must be specified.

Reviewer #1: Yes

Reviewer #2: Yes

---

4. Is the manuscript presented in an intelligible fashion and written in standard English?

PLOS ONE does not copyedit accepted manuscripts, so the language in submitted articles must be clear, correct, and unambiguous. Any typographical or grammatical errors should be corrected at revision, so please note any specific errors here.

Reviewer #1: Yes

Reviewer #2: Yes

---

5. Review Comments to the Author

Please use the space provided to explain your answers to the questions above. You may also include additional comments for the author, including concerns about dual publication, research ethics, or publication ethics. (Please upload your review as an attachment if it exceeds 20,000 characters)

Reviewer #1: This article by Song reports the fluctuations in the interfacial region between proteins, interpreted as a material property. The author draws an interesting analogy between the mechanical properties of fibrils and the interfacial mechanics of the proteins. The key point appears to be justified: the fluctuation of the interface between proteins is softer than the internal fluctuations of a protein because of the different interactions and degrees of freedom.

The key point missing from this paper is that the mechanical model is not truly required for looking at the fluctuations of proteins; they really are not isotropic materials. Discussion of this really must be done to make the point of the article.

Response: Thank you for the comment. It is true that a mechanical model is not necessary for studying the fluctuations of proteins. Indeed, such fluctuations were mostly studied by normal model analysis (NMA) in the past. However, mechanical models *can* be used to study the fluctuations (see Refs 21, 26 for example). By bridging between mechanical models with NMA, as is the focus of this paper, we can determine the elastic properties of proteins. Proteins have been modelled as isotropic elastic materials for decades; see most of the references cited in the Introduction. The reference provided by the reviewer near the end of this review is another example.

My comments below mostly complain about the naive analysis of the van der waals interactions between proteins which I think needs to be modified.

Major comments:

In my opinion normal mode analysis does not really qualify as predicting thermal vibrations because it is using the thermal vibrations to compute the modes. Casting it in terms of an elastic material does not change this. What the model can do is predict the strain in response to stress. Alternatively, the author might cast it as the way of predicting the thermal vibrations of large fibers from a local property of the dimer (abstract).

Response: the reviewer can be assured that normal model analysis, developed by pioneers such as Levitt, Karplus, and Go in the 1980's, is the best method for predicting thermal vibrations of proteins. NMA does not use thermal vibrations to compute modes. It is the other way around: It uses the Hessian matrix to compute modes, which in turn are used to predict the extent of thermal vibrations.

Proteins aren't mostly held together by van der waals forces. Here you must know that this could only refer to the fold of a protein. The atoms of a protein are connected by covalent bonds. The hydrophobic effect and hydrogen bonding are mostly at play. The hydrophobic effect is not the van der waals force (Section 1). "The interface region is kept together mostly by van der Waals interactions" (Section 3.5).

Model peptides and water near the interface region experience relatively strong Coulomb interactions with the protein and are repelled by the vdW approximation to the Pauli repulsion at close contact (the 1/r12 potential). I believe there would still be a fluctuating interface even in the complete absence of an attractive 1/r^6 potential. Therefore, the estimate is unjustified.

Response: Thank you for the comment. In this work when we speak about proteins, or proteins as solids, what we have in mind is always folded proteins. The primary structure of a protein is a 1D, linear sequence of amino acids that are connected by covalent bonds. Upon forming a 3-D tertiary structure, the number of covalent bonds remains unchanged and accounts for only a small percentage of the total number of interactions among the atoms in the 3D folded structure. Therefore, what holds a protein together in a 3-D structure is not covalent bonds, but <u>two major non-bonded interactions</u>: van der Waals interactions (non-polar interaction) and electrostatic interactions (polar interactions). Hydrogen bonds is not a fundamental interaction itself, but only a net result of electrostatic interactions and van der Waals interactions. This is the reason why in most standard force fields such as CHARMM, there is no hydrogen bond term. The hydrophobic effect, where non-polar residues clump together to minimize the disruption of polar interactions in water, is also a net result of van der Waals interactions and polar interactions.

Ideally, it is the best of include both van der Waals and electrostatic interactions in a model. However, electrostatic interactions are difficult to include in normal mode analysis since they introduce negative spring constants [Ref. 33]. Furthermore, our past studies showed that van der Waals interactions provide a greater contribution than electrostatics [Ref. 33] and our sbNMA model, which does not include electrostatic terms, maintains most of the accuracy of the classical NMA [Ref. 33].

We have revised sections 1 and 3.5 to make our description more accurate; see pages 2 and 21.

The fluctuating interface shown in Figure 3 is interesting but because it is a novel result and appears challenging to validate a more sophisticated estimate would be interesting to see. Is this interface the result of hydrophobic contacts? Can the fluctuations of the hydrophobic contact volume be explained in terms of the hydrophobic-effect-driven tension? This would seem to be a more fruitful explanation than purely in terms of vdW contacts.

Response: Thank you for the comment. Since protein surfaces tend to be polar, what primarily holds a dimer together is usually not hydrophobic effect, but hydrogen bonds. As mentioned above, hydrogen bonds are not a fundamental interaction itself, but only a net result of electrostatic interactions and van der Waals interactions. Electrostatic interactions are difficult to model in NMA and we will investigate possible ways to include them in future work. We have revised the text to explain this more clearly; for example, see page 21.

Figure 6 is unacceptable. Considering protein index is not important (x-axis) and the values per-protein are listed in a table, I suggest a plot of sbNMA Young's modulus vs. the B-factor young's modulus. For example, when the crystallographic Young's modulus (red) is greater than the sbNMA analysis (blue) the blue value is not shown. An XY/scatter plot would indicate the correlation of differences in protein Young's modulus.

Response: Thank you for the comment. The proteins are actually listed by their sizes in the ascending order as in Table 2. I have added a sentence in the caption to make this clear. The main purpose of the figure is to show the upper and lower bounds of Young's moduli, not their correlation. A scatter plot will not serve this purpose well. To address the problem you raised, I have changed the plot to show the values of the blue bars always.

Minor:

"Along with liquid, gas, and plasma, solid is one of the four states of matter." Wikipedia lists quite a few more such as Bose-Einstein condensates. There is no reason to make such obvious statements in a scientific article. I urge the author to be less didactic (Section 1).

Response: this is now modified; see page 2.

The bulk modulus is not the Young's modulus and the author confuses them in the introduction. The Young's modulus refers to strain along a direction with the two other directions unconstrained. Water is a fluid and as such has a zero Young's modulus (Section 1).

Response: this is now clarified; see page 3.

What is the rationale for using the mass-weighted averages as opposed to anything else? Mass has nothing to do with any elastic modulus; it is independent of mass-scaling (2.3).

Response: That is a good question. The mass-weighting is for computing the magnitude of fluctuations of a whole protein. Past study (see Ref. [32]) demonstrated that mass-weighting is physically much more meaningful than simply averaging as it leads to an elegant expression of the magnitude as a function of characteristic frequency ω_p (see Eq. (22) in Ref [32]).

Lessen et al, JCTC 2018 is another computational study attempting to compute the Young's modulus: https://dx.doi.org/10.1021/acs.jctc.8b00377

Response: Thank you for reference! It is now added to the Introduction.

Reviewer #2: This manuscript discusses calculations and results on the elasticity of proteins and their interfaces. The underlying ideas is to equate the results of calculations based on solid elastic models with those based on atomic vibrations either from atomistic MD simulations or crystallographic temperature factors. The issue of elasticity of proteins and their interfaces is an interesting one, and the basic mathematical idea is sound and well executed. There is however a very important problem with the paper with regard to how the author has chosen to think about protein interfaces, perhaps reflecting some unfamiliarity with how structural biologists think about protein quaternary structure vs incidental crystal contacts. Basically, the underlying idea of the paper is interesting and perhaps informative, but to be meaningful the study would have to be repeated with a different set of classifications of what is considered a protein interface.

Major problem:
The author takes crystal structures from the PDB and generates protein interfaces by examining additional chains that are in contact based on crystallographic operations. By itself, this is not an appropriate procedure for defining protein interfaces. The study doesn't make the critical distinction between real protein-protein interfaces (i.e. those that have arisen in nature by evolution) and fortuitous contacts (often very small) that occur incidentally during crystallization. The former category is of substantial interest. The latter category might also be of interest in a more specialized context. But it is vital that the two things not be confused or conflated. There is a long literature on both subjects. There are also dedicated databased set up to help disentangle the two cases, which is not always easy. The PISA protein database is one. The PQS protein quaternary structure database is another.

Just to be more explicit, one could go to such a resource and ask for a list of PDB structures where the protein molecule is believed to be a monomer in its native form (comprised of just one protein chain); for those cases, contacts present in the expanded crystal state would (presumably) represent fortuitous protein-protein crystal contacts. For analyzing natural protein-protein interfaces, one can query the database for proteins believed to exist as dimers in their natural biological forms. Then one can extract the dimeric forms directly from that curated database – obtaining the correct biological forms is not always trivial to do by expanding from the coordinates in the PDB as the author has attempted to do based on closest contact. Analysis of these cases would then represent evolved protein-protein interfaces. And of course quaternary structures higher than dimers could be considered, with added complexity. Once the author does this, then the results, which are likely to differ substantially, can be analyzed in separate categories of natural and incidental crystal interfaces. It is a virtual certainty that the cases of natural interfaces will not be as flexible as the whole set of protein-protein contacts (including incidental crystal contacts) which the author has presently defined.

Response: this is an excellent comment! We have added another set of 18 high resolution (1.0 A or better) structures that are naturally dimers (with C2 symmetry and are in the dimer form in the asymmetric unit). The results are just as you predicted. The nature interfaces are significantly stiffer. I have thoroughly revision section 3.6 to include the new results and have modified the discussion accordingly.

Minor points:
1) At the bottom of page 8, the form and units of the stiffness matrix K should be given. E.g. is it the collection of second partial derivatives of the conformational energy (normalized by kT) with respect to atomic coordinates (or finite elements)? Similarly for the Hessian at the top of page 10.

Response: The Hessian matrix is a 3n-by-3n matrix containing the second partial derivatives of the potential energy function with respect to the 3n atomic coordinates. The global stiffness matrix K is built with element stiffness matrices for all the elements (tetrahedral in the current paper). Each element stiffness matrix is 12x12 matrix and can be written as:

k = [B]^T [D] [B] V

where B and D are standard matrices available in standard textbooks on finite element method that depend solely on the coordinates of the four nodes of the tetrahedral element, and V is the volume of the tetrahedron. The unit of both stiffness and Hessian matrices is kcal/mol/A^2.

2) The legend to Figure 4 should clarify that the two kinds of experimental results are being interpreted as upper and lower bounds.

Response: done. Thank you.

3) The second paragraph on page 11 should introduce, at least parenthetically, that the accepted technical term for 'B-factors' is 'atomic displacement parameters'.

Response: Thank you for the suggestion. It is added now.

4) Protein-protein interfaces are customarily discussed/plotted with respect to buried surface area rather than volumes.

Response: Thank you for the great suggestion. We have replaced the volume information with buried surface areas throughout.

---

6. PLOS authors have the option to publish the peer review history of their article (what does this mean?). If published, this will include your full peer review and any attached files.

If you choose "no", your identity will remain anonymous but your review may still be made public.

Do you want your identity to be public for this peer review? For information about this choice, including consent withdrawal, please see our Privacy Policy.

Reviewer #1: No

Reviewer #2: No

[NOTE: If reviewer comments were submitted as an attachment file, they will be attached to this email and accessible via the submission site. Please log into your account, locate the manuscript record, and check for the action link "View Attachments". If this link does not appear, there are no attachment files.]

While revising your submission, please upload your figure files to the Preflight Analysis and Conversion Engine (PACE) digital diagnostic tool, https://pacev2.apexcovantage.com/. PACE helps ensure that figures meet PLOS requirements. To use PACE, you must first register as a user. Registration is free. Then, login and navigate to the UPLOAD tab, where you will find detailed instructions on how to use the tool. If you encounter any issues or have any questions when using PACE, please email PLOS at figures@plos.org. Please note that Supporting Information files do not need this step.
